# Supplementary material for: Omicron variant neutralizing antibodies following BNT162b2 BA.4/5 versus mRNA-1273 BA.1 bivalent vaccination in patients with end-stage kidney disease
Source: Nat Commun. 2023 Sep 27;14:6041. doi: 10.1038/s41467-023-41678-9 (PMC10533557; doi:10.1038/s41467-023-41678-9)
Supplement: Supplementary file 1 — Supplementary Information [file 41467_2023_41678_MOESM1_ESM.pdf]

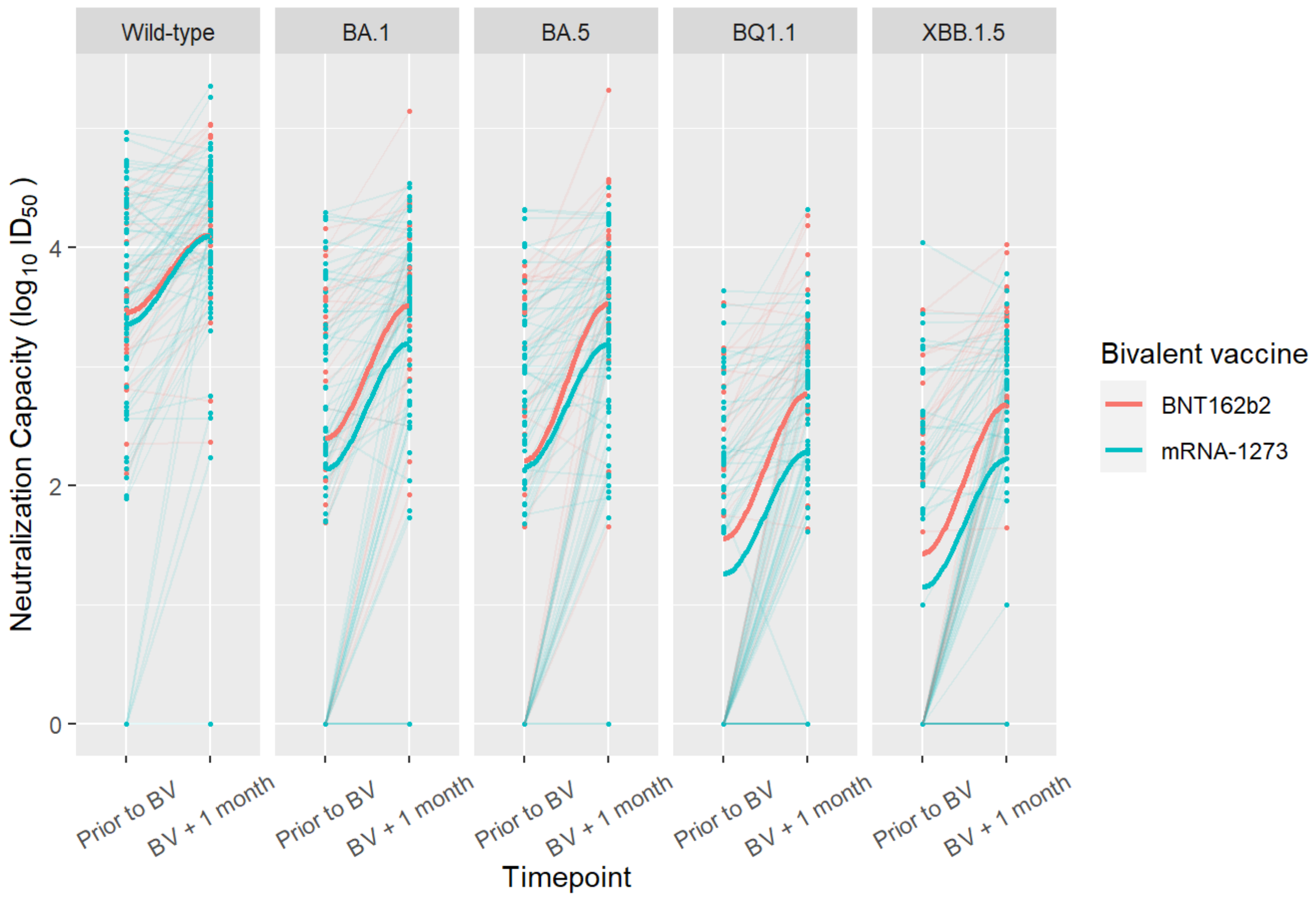

Supplementary Fig 1 | Neutralizing antibody trajectories for wild-type, BA.1, BA.5, BQ.1.1, and XBB.1.5 subvariants prior to and one month following bivalent vaccination with BNT162b2 BA.4/5 (n=26) or mRNA-1273 BA.1 (n=72). Differences by vaccine type were not statistically different after adjustment for baseline neutralizing antibody levels, anti-nucleocapsid positivity, patient type, and number of vaccine doses: Wild-type (P=0.48), BA.1 (P=0.21), BA.5 (P=0.069), BQ.1.1 (P=0.10), nor XBB.1.5 (P=0.099). Dots represent individual serum samples collected (n=98 for each time point). Results were analysed using a linear mixed effects model, with a two-sided p-value. No adjustments were made for multiple comparisons.

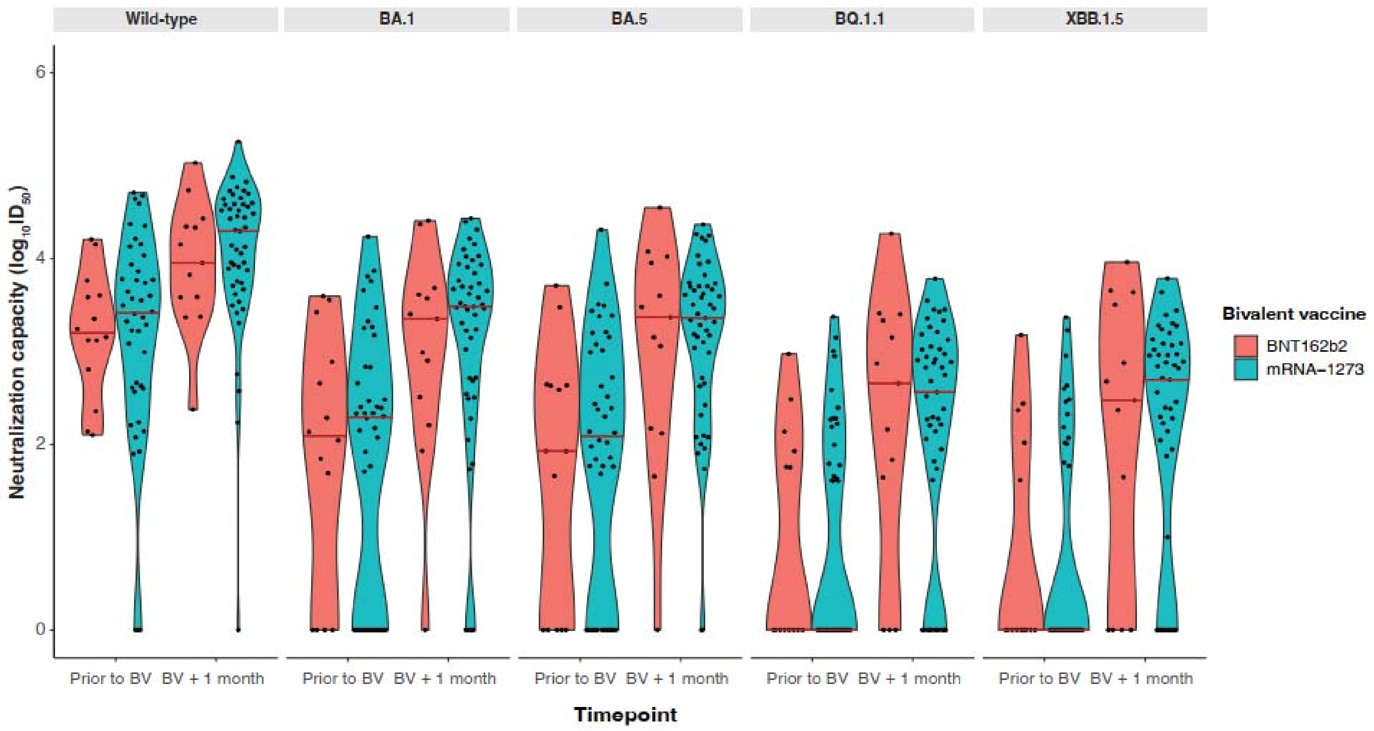

Supplementary Fig 2 | Neutralizing antibodies against wild-type, BA.1, BA.5, BQ.1.1, and XBB.1.5 by bivalent vaccine type in hemodialysis patients: BNT162b2 BA.4/5 (n=14) versus mRNA-1273 (n=44) after exclusion of participants with a positive anti-nucleocapsid antibody. Solid red line indicates median level. Dots represent individual serum samples collected (n=58). Results were analysed using a linear mixed effects model, with a two-sided p-value. No adjustments were made for multiple comparisons.

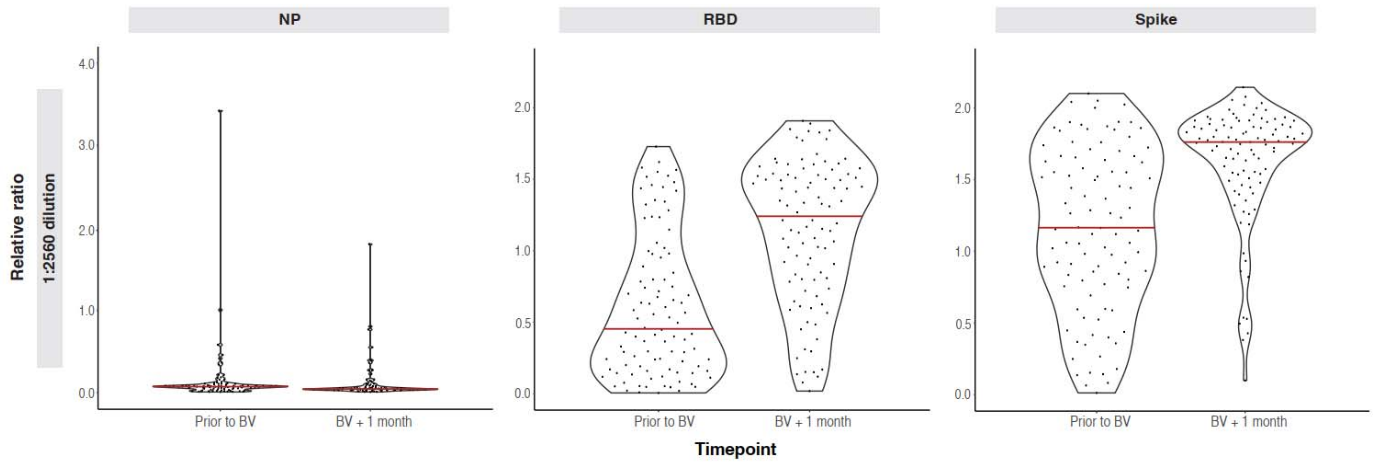

Supplementary Fig 3 | SARS-CoV-2 IgG nucleocapsid, RBD and Spike Binding Antibody Response. Dots represent individual serum samples collected (n=98 for each timepoint). Solid red line indicates median level. Antibody levels are reported as relative ratios to synthetic standards. Upper limit of the linear range for the relative ratio is 2 for nucleocapsid, 1.2 for RBD and 1.2 for Spike. Anti-RBD increased from 0.45 (IQR 0.18, 0.97) to 1.24 (IQR 0.80, 1.53) 1-month post-vaccination and anti-spike increased from 1.16 (IQR 0.77, 1.65) to 1.76 (IQR 1.52,1.86) 1-month post-vaccination ($p<0.0001$ for difference prior and 1 month following bivalent vaccination). Abbreviations: BV, bivalent; N, nucleocapsid protein; RBD, receptor binding domain.

Supplementary Table 1 | Anti-nucleocapsid IgG seropositivity and seroconversion prior to and 1 month following bivalent vaccination.

Timepoint	Anti-Nucleocapsid IgG Seropositivity
Pre-Bivalent	40/98 (41%)
Bivalent Vaccine + 1 month	36/98 (37%)

At follow-up, one seroconversion for anti-nucleocapsid IgG occurred and 5 participants initially seropositive for anti-nucleocapsid IgG became seronegative.

Supplementary Table 2 | Neutralizing and total antibody levels in overall cohort prior to and following bivalent vaccination.

Variable	Pre-Bivalent (n= 98)*	Post-Bivalent (n= 98)	Overall (n= 196)
**Wild-type neutralization**	93 (95%)	97 (99%)	190 (97%)
**Wild-type $\log_{10}$ ID50**	3·59 (2·99, 4·15)	4·25 (3·81, 4·54)	3·93 (3·37, 4·42)
**BA.1 neutralization**	74 (76%)	91 (93%)	165 (84%)
**BA.1 $\log_{10}$ ID50**	2·37 (1·69, 3·35)	3·59 (2·92, 3·98)	3·23 (2·19, 3·76)
**BA.5 neutralization**	73 (74%)	94 (96%)	167 (85%)
**BA.5 $\log_{10}$ ID50**	2·61 (0·41, 3·20)	3·53 (3·00, 3·92)	3·13 (2·09, 3·70)
**BQ.1.1 neutralization**	54 (55%)	82 (84%)	136 (69%)
**BQ.1.1 $\log_{10}$ ID50**	1·70 (0·00, 2·38)	2·86 (2·06, 3·23)	2·23 (0·00, 2·98)
**XBB.1.5 neutralization**	47 (48%)	79 (81%)	126 (64%)
**XBB.1.5 $\log_{10}$ID50**	0·00 (0·00, 2·45)	2·83 (2·05, 3·24)	2·28 (0·00, 3·02)
**Anti-RBD IgG, relative ratio**	0·45 (0·18, 0·97)	1·24 (0·80, 1·53)	0·84 (0·33, 1·44)
**Anti-Spike IgG, relative ratio**	1·16 (0·77, 1·65)	1·76 (1·52, 1·86)	1·56 (1·01, 1·81)
**Anti-N positive**	40 (41%)	36 (37%)	76 (39%)
*Median (IQR); n (%)			

Abbreviations: Anti-N, anti-nucleocapsid, anti-RBD, anti-receptor binding domain; Relative ratios above 1·2 may be underestimated due to assay saturation.

Supplementary Table 3 | Fold decrease in neutralization in comparison to wild-type (D614G)

	Pre-Bivalent mRNA-1273 (n=72)	Post Bivalent mRNA-1273 (n=72)	Pre-Bivalent BNT162b2 (n=26)	Post Bivalent BNT162b2 (n=26)	Total (n=196)
**Median BA.1 Fold Decrease [IQR]***	12.2 (60.4)	5.58 (9.3)	7.2 (31.7)	3.74 (5.1)	7.3 (17.5)
**Median BA.5 Fold Decrease [IQR]**	12.8 (37.9)	7.6 (12.4)	9.1 (99.1)	3.1 (5.1)	8.3 (19.2)
**Median BQ.1.1 Fold Decrease [IQR]**	85.4 (436.4)	39.0 (92.1)	45.5 (510.4)	17.6 (25.5)	45.8 (170.3)
**Median XBB.1.5. Fold Decrease [IQR]**	108.3 (1282.7)	42.3 (60.5)	74.5 (1140.6)	18.7 (25.9)	48.2 (376.7)

* Fold changes are expressed in comparison to wild-type (D614G) ancestral SARS-CoV-2.

Supplementary Table 4 | Neutralizing antibody capacity against wild-type, B.1.1.529 Omicron subvariants following bivalent mRNA COVID-19 vaccination by bivalent vaccine type.

Variable	Overall (n = 98)*	mRNA-1273 (n= 72)*	BNT162b2 (n= 26)*
**Wild-type neutralization**	97 (99%)	71 (99%)	26 (100%)
**Wild-type $\log_{10}$ ID50**	4.25 (3.81, 4.54)	4.25 (3.81, 4.56)	4.22 (3.85, 4.43)
**BA.1 neutralization**	91 (93%)	66 (92%)	25 (96%)
**BA.1 $\log_{10}$ ID50**	3.59 (2.92, 3.98)	3.54 (2.78, 3.93)	3.73 (3.13, 4.16)
**BA.5 neutralization**	94 (96%)	69 (96%)	25 (96%)
**BA.5 $\log_{10}$ ID50**	3.53 (3.00, 3.92)	3.48 (2.72, 3.73)	3.80 (3.35, 4.08)
**BQ.1.1 neutralization**	82 (84%)	59 (82%)	23 (88%)
**BQ.1.1 $\log_{10}$ ID50**	2.86 (2.06, 3.23)	2.71 (1.99, 3.12)	3.16 (2.62, 3.41)
**XBB.1.5 neutralization**	79 (81%)	57 (79%)	22 (85%)
**XBB.1.5 $\log_{10}$ID50**	2.83 (2.05, 3.24)	2.69 (1.93, 3.13)	3.16 (2.52, 3.49)
**COVID-19 vaccine dose number**			
4	8 (8.2%)	6 (8.3%)	2 (7.7%)
5	90 (92%)	66 (92%)	24 (92%)
**Prior COVID-19**	25 (26%)	19 (26%)	6 (23%)
* n (%); Median (IQR)			

Supplementary Table 5 | Neutralizing antibody response differences between BNT162b2 BA.4/5 vaccine and mRNA-1273 BA.1 vaccine while accounting for initial two dose vaccine type, third vaccine dose type, anti-nucleocapsid status, number of vaccine doses, patient type, and anti-nucleocapsid positivity

Variant of Concern	p-value
Wild-Type	0.26
BA.1	0.49
BA.5	0.23
BQ.1.1	0.50
XBB.1.5	0.39

Supplementary Table 6 | Neutralizing antibody capacity against wild-type, B.1.1.529 Omicron subvariants following bivalent mRNA COVID-19 vaccination by number of total vaccine doses.

Variable	Overall (n= 98)*	Four Doses (n=8)*	Five Doses (n=90)*
**Wild-type neutralization**	97 (99%)	8 (100%)	89 (99%)
**Wild-type log_{10} ID50**	4.25 (3.81, 4.54)	4.19 (3.94, 4.26)	4.28 (3.81, 4.55)
**BA.1 neutralization**	91 (93%)	8 (100%)	83 (92%)
**BA.1 log_{10} ID50**	3.59 (2.92, 3.98)	3.74 (3.40, 4.16)	3.59 (2.89, 3.97)
**BA.5 neutralization**	94 (96%)	8 (100%)	86 (96%)
**BA.5 log_{10} ID50**	3.53 (3.00, 3.92)	3.60 (3.34, 4.08)	3.53 (2.94, 3.90)
**BQ.1.1 neutralization**	82 (84%)	7 (88%)	75 (83%)
**BQ.1.1 log_{10} ID50**	2.86 (2.06, 3.23)	2.93 (2.72, 3.33)	2.82 (2.02, 3.21)
**XBB.1.5 neutralization**	79 (81%)	7 (88%)	72 (80%)
**XBB.1.5 log_{10}ID50**	2.83 (2.05, 3.24)	2.72 (2.57, 3.32)	2.84 (1.97, 3.23)
**Bivalent Vaccine Type**			
mRNA-1273	72 (73%)	6 (75%)	66 (73%)
BNT162b2	26 (27%)	2 (25%)	24 (27%)
**Patient Type**			
HD	83 (85%)	8 (100%)	75 (83%)
Kidney Transplant	15 (15%)	0 (0%)	15 (17%)
**Prior COVID-19**	25 (26%)	5 (62%)	20 (22%)

* n (%); Median (IQR)

Abbreviations: HD, hemodialysis.

Supplementary Table 7 | Neutralizing antibody capacity against wild-type, B.1.1.529 Omicron subvariants following bivalent mRNA COVID-19 vaccination by COVID-19 status.

Variable	Overall (n = 98)*	No COVID-19 (n= 73)*	Prior COVID-19 (n=25)*
**Wild-type neutralization**	97 (99%)	73 (100%)	24 (96%)
**Wild-type log_{10} ID50**	4.25 (3.81, 4.54)	4.27 (3.79, 4.59)	4.22 (3.93, 4.42)
**BA.1 neutralization**	91 (93%)	67 (92%)	24 (96%)
**BA.1 log_{10} ID50**	3.59 (2.92, 3.98)	3.52 (2.71, 3.93)	3.82 (3.51, 4.00)
**BA.5 neutralization**	94 (96%)	69 (95%)	25 (100%)
**BA.5 log_{10} ID50**	3.53 (3.00, 3.92)	3.37 (2.67, 3.88)	3.70 (3.53, 3.95)
**BQ.1.1 neutralization**	82 (84%)	58 (79%)	24 (96%)
**BQ.1.1 log_{10} ID50**	2.86 (2.06, 3.23)	2.68 (1.74, 3.18)	3.03 (2.63, 3.29)
**XBB.1.5 neutralization**	79 (81%)	56 (77%)	23 (92%)
**XBB.1.5 log_{10}ID50**	2.83 (2.05, 3.24)	2.71 (1.65, 3.24)	3.02 (2.56, 3.30)
**Dose Bivalent Vaccine**			
4	8 (8.2%)	3 (4.1%)	5 (20%)
5	90 (92%)	70 (96%)	20 (80%)
**Bivalent Vaccine Type**			
mRNA-1273	72 (73%)	53 (73%)	19 (76%)
BNT162b2	26 (27%)	20 (27%)	6 (24%)

* n (%); Median (IQR)

Supplementary Table 8 | Neutralizing antibody capacity against wild-type, B.1.1.529 (Omicron subvariants following bivalent mRNA COVID-19 vaccination by patient type.

Variable	Overall<br>(n= 98)*	HD<br>(n= 83)*	Kidney Transplant<br>(n=15)*
**Wild-type neutralization**	97 (99%)	82 (99%)	15 (100%)
**Wild-type $\log_{10}$ ID50**	4.25 (3.81, 4.54)	4.27 (3.79, 4.57)	4.10 (3.84, 4.38)
**BA.1 neutralization**	91 (93%)	78 (94%)	13 (87%)
**BA.1 $\log_{10}$ ID50**	3.59 (2.92, 3.98)	3.65 (3.11, 4.00)	3.15 (2.44, 3.63)
**BA.5 neutralization**	94 (96%)	80 (96%)	14 (93%)
**BA.5 $\log_{10}$ ID50**	3.53 (3.00, 3.92)	3.59 (3.11, 3.95)	3.13 (2.61, 3.52)
**BQ.1.1 neutralization**	82 (84%)	73 (88%)	9 (60%)
**BQ.1.1 $\log_{10}$ ID50**	2.86 (2.06, 3.23)	2.91 (2.16, 3.27)	2.21 (0.00, 2.75)
**XBB.1.5 neutralization**	79 (81%)	69 (83%)	10 (67%)
**XBB.1.5 $\log_{10}$ID50**	2.83 (2.05, 3.24)	2.88 (2.29, 3.28)	2.06 (0.00, 2.76)
**Dose Bivalent Vaccine**			
4	8 (8.2%)	8 (9.6%)	0 (0%)
5	90 (92%)	75 (90%)	15 (100%)
**Bivalent Vaccine Type**			
mRNA-1273	72 (73%)	57 (69%)	15 (100%)
BNT162b2	26 (27%)	26 (31%)	0 (0%)
**Prior COVID-19**	25 (26%)	23 (28%)	2 (13%)
* n (%); Median (IQR)			

Abbreviations: HD, hemodialysis.
