# Supplementary material for: Omicron variant neutralizing antibodies following BNT162b2 BA.4/5 versus mRNA-1273 BA.1 bivalent vaccination in patients with end-stage kidney disease
Source: Nat Commun. 2023 Sep 27;14:6041. doi: 10.1038/s41467-023-41678-9 (PMC10533557; doi:10.1038/s41467-023-41678-9)
Supplement: Supplementary file 3 — Reporting Summary [file 41467_2023_41678_MOESM3_ESM.pdf]

nature portfolio

Corresponding author(s): Michelle Hladunewich

Last updated by author(s): Sep 11, 2023

Reporting Summary

Nature Portfolio wishes to improve the reproducibility of the work that we publish. This form provides structure for consistency and transparency in reporting. For further information on Nature Portfolio policies, see our Editorial Policies and the Editorial Policy Checklist.

Statistics

For all statistical analyses, confirm that the following items are present in the figure legend, table legend, main text, or Methods section.

n/a	Confirmed	
☐	☒	The exact sample size (*n*) for each experimental group/condition, given as a discrete number and unit of measurement
☐	☒	A statement on whether measurements were taken from distinct samples or whether the same sample was measured repeatedly
☐	☒	The statistical test(s) used AND whether they are one- or two-sided<br>*Only common tests should be described solely by name; describe more complex techniques in the Methods section.*
☐	☒	A description of all covariates tested
☐	☒	A description of any assumptions or corrections, such as tests of normality and adjustment for multiple comparisons
☐	☒	A full description of the statistical parameters including central tendency (e.g. means) or other basic estimates (e.g. regression coefficient) AND variation (e.g. standard deviation) or associated estimates of uncertainty (e.g. confidence intervals)
☐	☒	For null hypothesis testing, the test statistic (e.g. *F*, *t*, *r*) with confidence intervals, effect sizes, degrees of freedom and *P* value noted<br>*Give P values as exact values whenever suitable.*
☒	☐	For Bayesian analysis, information on the choice of priors and Markov chain Monte Carlo settings
☒	☐	For hierarchical and complex designs, identification of the appropriate level for tests and full reporting of outcomes
☒	☐	Estimates of effect sizes (e.g. Cohen's *d*, Pearson's *r*), indicating how they were calculated

Our web collection on statistics for biologists contains articles on many of the points above.

Software and code

Policy information about availability of computer code

Data collection	Data was processed using R version 4.0.5.
Data analysis	Data was analysed using code written in R version 4.0.5 (R Project for Statistical Computing).<br>GraphPad Prism version 9.4.1

For manuscripts utilizing custom algorithms or software that are central to the research but not yet described in published literature, software must be made available to editors and reviewers. We strongly encourage code deposition in a community repository (e.g. GitHub). See the Nature Portfolio guidelines for submitting code & software for further information.

Data

Policy information about availability of data

All manuscripts must include a data availability statement. This statement should provide the following information, where applicable:

- Accession codes, unique identifiers, or web links for publicly available datasets
- A description of any restrictions on data availability
- For clinical datasets or third party data, please ensure that the statement adheres to our policy

The serologic data generated in this study are openly available through the publicly accessible Borealis repository (DOI: https://doi.org/10.5683/SP3/9XUY6O). The

patient-specific clinical data are available under restricted access through the publicly accessible CITF Databank. The source data generated in this study for figures are provided in the Supplementary Information/Source Data file.

Human research participants

Policy information about studies involving human research participants and Sex and Gender in Research.

Reporting on sex and gender	Information on sex was collected from clinical records. Information on gender was not collected. The overall number of participants who were male or female were reported. No sex-based analyses were performed given the sample size.
Population characteristics	All participants were receiving dialysis or had a functional kidney transplant. Only participants unable to provide informed consent due to cognitive impairment or a language barrier if a translator was unavailable were excluded. The median age was 70 years and 35% of participants were female.
Recruitment	Patients were recruited in-person at dialysis treatment or at clinic visits or via telephone. This was a prospective observational study and all participants provided written informed consent. The study is reflective of the dialysis and kidney transplant populations receiving COVID-19 vaccination.
Ethics oversight	This study protocol was approved by the respective Institutional Review Boards at Sunnybrook Health Sciences Centre and Unity Health Network (CTO #3604) as well as Michael Garron Hospital (REB # 856-2201-Inf-066).

Note that full information on the approval of the study protocol must also be provided in the manuscript.

Field-specific reporting

Please select the one below that is the best fit for your research. If you are not sure, read the appropriate sections before making your selection.

☒ Life sciences ☐ Behavioural & social sciences ☐ Ecological, evolutionary & environmental sciences

For a reference copy of the document with all sections, see nature.com/documents/nr-reporting-summary-flat.pdf

Life sciences study design

All studies must disclose on these points even when the disclosure is negative.

Sample size	No formal sample size calculation was performed. This study was part of a larger observational study which recruited 2236 participants with kidney disease to evaluate serologic response to vaccination. We selected a convenience sample of n=100 based upon available participants who had received bivalent vaccines and reasonable capacity to perform the serologic testing.
Data exclusions	Only participants unable to provide informed consent due to cognitive impairment or a language barrier if a translator is unavailable were excluded.
Replication	Binding and neutralizing antibody assays used are described previously in https://onlinelibrary.wiley.com/doi/full/10.1002/cti2.1380 and https://insight.jci.org/articles/view/142362. All assays have been previously published by our group in peer-reviewed journals. No sample replicates were performed due to limited sample material however there were 98 unique samples tested prior to bivalent vaccination and 98 samples tested following bivalent vaccination.
Randomization	Not applicable. Allocation to the study vaccine was not randomized as this was an observational study. We adjusted for the following pre-specified covariates in multivariable mixed effects models: vaccine time point (pre-bivalent versus bivalent vaccine + 1 month, bivalent vaccine type, number of COVID-19 vaccine doses (four versus five), kidney transplant recipients versus dialysis patients, and anti-nucleocapsid antibody status.
Blinding	This was an observational study rather than a randomized controlled trial where patients had already received their vaccine in routine clinical care, therefore they were aware of the vaccine type they had received and blinding was not possible.

Reporting for specific materials, systems and methods

We require information from authors about some types of materials, experimental systems and methods used in many studies. Here, indicate whether each material, system or method listed is relevant to your study. If you are not sure if a list item applies to your research, read the appropriate section before selecting a response.

Materials & experimental systems

n/a	Involved in the study	
☐	☒	Antibodies
☐	☒	Eukaryotic cell lines
☒	☐	Palaeontology and archaeology
☒	☐	Animals and other organisms
☒	☐	Clinical data
☒	☐	Dual use research of concern

Methods

n/a	Involved in the study	
☒	☐	ChIP-seq
☒	☐	Flow cytometry
☒	☐	MRI-based neuroimaging

Antibodies

Antibodies used

Anti-human IgG#5 secondary antibody fused to horseradish peroxidase (HRP); Source: Colwill et al. Clinical & Translational Immunology 2022; Identifier: IgG#5-HRP
Anti-RBD IgG antibody VHH72; Source: Colwill et al. Clinical & Translational Immunology 2022; Identifier: VHH72-hFc1X7
Anti-nucleocapsid IgG antibody HC2003; Source: Genscript; Identifier: Cat#A02039

Validation

Anti-RBD and anti-nucleocapsid primary antibodies were validated using purified RBD and nucleocapsid as described in Colwill et al., Clinical & Translational Immunology 2022. Anti-nucleocapsid IgG was obtained from a commercial vendor who also validated its specificity to nucleocapsid.

Eukaryotic cell lines

Policy information about cell lines and Sex and Gender in Research

Cell line source(s)

The HEK293T-ACE2/TMPRSS2 stable cell pool was generated as described in Abe et al, JCI Insight 2020, followed by Western blotting to verify the expressions of ACE2 and TMRESS2 using commercial antibodies.

Authentication

Cell lines were authenticated by the provider.

Mycoplasma contamination

Cell lines were tested for Mycoplasma (and were negative by PCR).

Commonly misidentified lines
(See ICLAC register)

No commonly misidentified cell lines were used.
